# Simulator training for enhanced interventional radiology education

**DOI:** 10.1038/s41598-025-95828-8

**Published:** 2025-03-29

**Authors:** Nina Kronner, Markus Strebl, Julian Lenk, Bernhard Gebauer, Oliver Schweizerhof, Anne Frisch, Charlotte Wintergerst, Wibke Uller, Maximilian de Bucourt, Elif Can

**Affiliations:** 1 Department of Radiology, Charité - Universitätsmedizin Berlin, Hindenburgdamm 30, 12203 Berlin, Germany; 2 Institute of Biometry and Clinical Epidemiology, Charité – Universitätsmedizin Berlin, Charitéplatz 1, 10117 Berlin, Germany; 3 Berlin Institute of Health, Charité – Universitätsmedizin Berlin, Charitéplatz 1, 10117 Berlin, Germany; 4 Department of Diagnostic and Interventional Radiology, Medical Center – University of Freiburg, Faculty of Medicine, University of Freiburg, Freiburg, Germany; 5 Corporate Member of Freie Universität Berlin and Humboldt- Universität zu Berlin, Department of Diagnostic and Interventional Radiology, Charité - Universitätsmedizin Berlin, Augustenburger Platz 1, 13353 Berlin, Germany; 6 Charité - Universitätsmedizin Berlin, Charitéplatz 1, 10117 Berlin, Germany

**Keywords:** Medical education, Interventional radiology, Virtual reality (VR), Simulator training, VR-enhanced simulation, Medical training outcomes, Health care, Medical research

## Abstract

To address the challenges of staff shortages and the need to gain practical experience in interventional radiology by increasing attention in the medical curriculum, especially in combination with the opportunity to successfully gain hands-on experience, can help influence medical students’ career decisions in favor of IR. Regular training on VR simulators can reduce the amount of X-ray radiation needed to adequately care for patients. Ten medical students underwent five angiographic training sessions using an endovascular simulator. Virtual fluoroscopy time was recorded during each session to measure skill development. Pre- and post-training questionnaires were conducted to assess changes in subjective proficiency and career interests. The median virtual fluoroscopy time decreased from 19.3 min initially to 9.3 min (*p* = 0.007), indicating enhanced procedural proficiency. Post-training questionnaires revealed a notable increase in interest in interventional radiology among participants. Additionally, participants reported improvements in practical skills, understanding of interventional radiology, and readiness for real-world interventions. Simulator-based training significantly enhances procedural proficiency and could impact career interests in interventional radiology. Despite the small sample size, the findings support the efficacy of VR training in medical education, highlighting the need for further research to optimize the implementation of simulation technology in medical training.

## Introduction

Interventional radiology (IR), a crucial yet underrepresented field in medical education, grapples with a significant shortfall in hands-on experience opportunities for students. Over the past 5 decades, virtual reality (VR) has emerged as a revolutionary tool in medical training, offering unparalleled benefits such as repetitive practice on simple tasks, cost reduction, and minimized surgical errors^1^. Despite its advantages and the growing need and interest in minimal invasive procedures, IR remains inadequately integrated in university curricula as a relatively young field. This insufficient coverage leads to a general unawareness among medical students. The gap contributes to a critical shortage of interventional radiologists, with 45% of positions unfilled in the UK in 2019 and nearly half of practitioners unable to provide round-the-clock services^2,3^. This scenario not only strains patient care but also contributes to a burnout prevalence of approximately 44% among European interventional radiologists as of 2023^4^.

Traditionally, the development of hands-on skills in medical training has occurred directly on patients, a method fraught with time consumption and inherent risks. Simulators present a safer alternative, allowing trainees to repeatedly practice complex procedures until they become second nature. Guided by mentors, students can receive tailored feedback, significantly enhancing their performance quality^5^. A comprehensive meta-analysis spanning 2 decades has shown that simulation-based medical education with deliberate practice yields superior clinical skill acquisition compared to traditional methods^6^.

Adult learning theories, particularly experiential learning, underscore the importance of concrete experience and active experimentation for long-term retention of knowledge. This approach is invaluable in medical education, where focusing on specific competences and skills is paramount^7,8^. High-quality realistic simulators, especially those enhanced by VR, have the potential to revolutionize learning processes in interventional radiology and beyond. These state-of-the-art tools provide a safe, controlled environment for learners to practice intricate procedures without the ethical dilemmas associated with patient safety. Such an environment not only hones technical skills but also influences career choices, potentially sparking a deeper interest in IR among students.

The implications of integrating advanced simulation technology into medical education are profound. By bridging the gap between theoretical learning and practical application, simulators facilitate a smoother transition for students from academia to the demanding realities of clinical practice. This transition, addressing both skill development and emotional resilience, is crucial for novice physicians^9,10^.

Recognizing these advantages, the BeST (Centre of Training and Simulation Berlin) was significantly upgraded and reopened at Charité-Campus Mitte in June 2024. This study aims to demonstrate the progress novice students can achieve through a series of five VR-enhanced simulator training sessions. We anticipate a marked increase in proficiency with catheter manipulation, evidenced by a reduction in virtual fluoroscopy time during simulated procedures. Moreover, we hypothesize that hands-on practice will not only reinforce theoretical knowledge but also enhance practical skills, fostering a deeper interest in radiology, particularly interventional radiology.

This paper explores the potential of simulation technology at the university level, particularly in fostering students’ interest and preparing them for angiographic interventions. The adoption of VR-enhanced simulators could herald a new era in medical education, improving patient care and educational outcomes.

## Materials and methods

### Participants

**Fig. 1:**
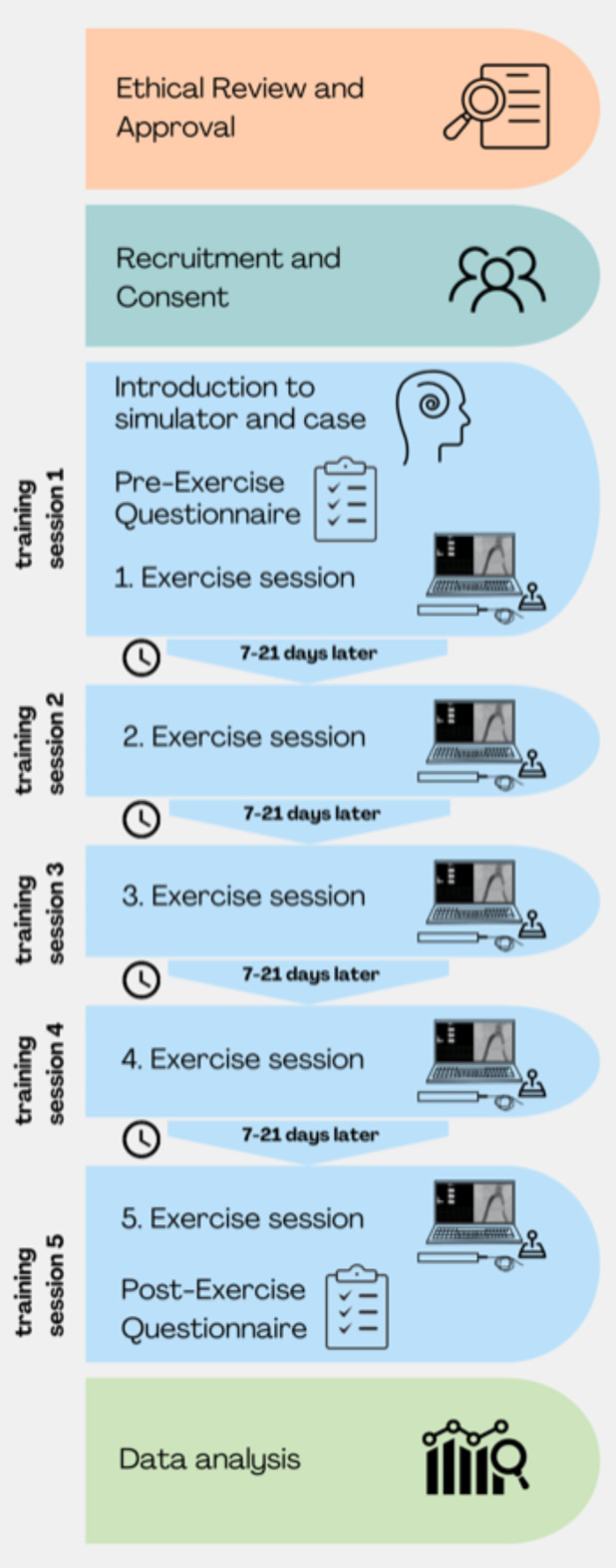
Flowchart of a Structured Training Program with Simulation and Case Study, Followed by Data Analysis. This flowchart outlines the steps of a structured training program designed for simulation and case study, including subsequent data analysis. The process is divided into several key phases.

This exploratory study prospectively collected data from ten voluntarily enrolled medical students at various stages of their education (second to sixth year) at a university. The number of participants was chosen based on the standard size of a regular university course. Recruitment occurred through an online leaflet following ethical committee approval. Participants were enrolled without prior practical experience in IR or IR simulators (see Fig. 1 for a flowchart illustrating participant selection and involvement). The participants completed five simulator training sessions willingly starting from a uniform knowledge baseline. All participants received an identical introduction to the simulator and its technical devices. Additionally, they were shown how to execute the case immediately before their first exercise session.

### Training procedure

**Fig. 2:**
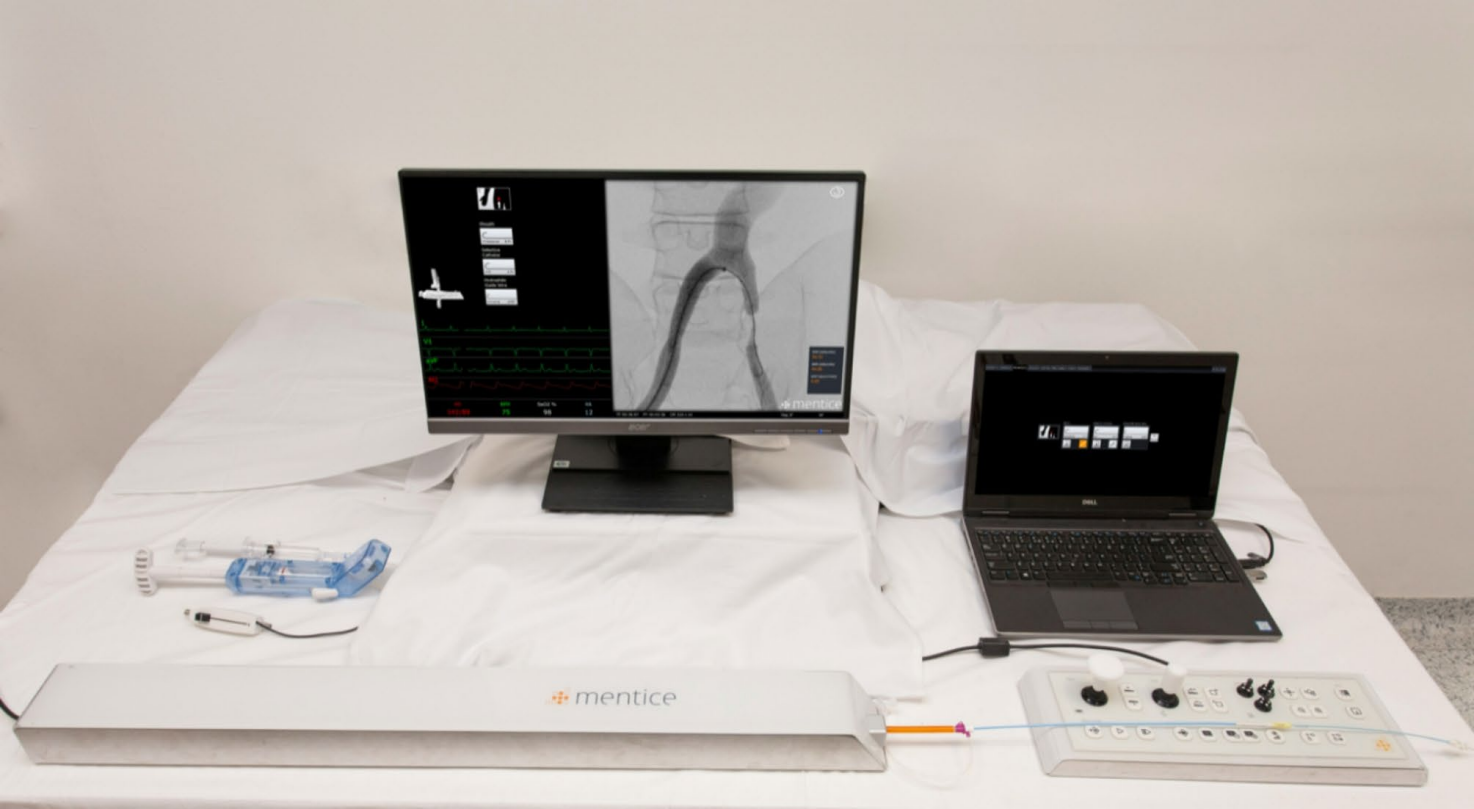
Experimental setup for the simulator training sessions, including the VIST G5 Mentice endovascular simulator, control joysticks, 3D monitor, fluoroscopy, CINE pedals, and device insertion model. This figure illustrates the comprehensive simulation environment used to emulate a catheter lab, enhancing the training experience for the participants.

**Fig. 3:**
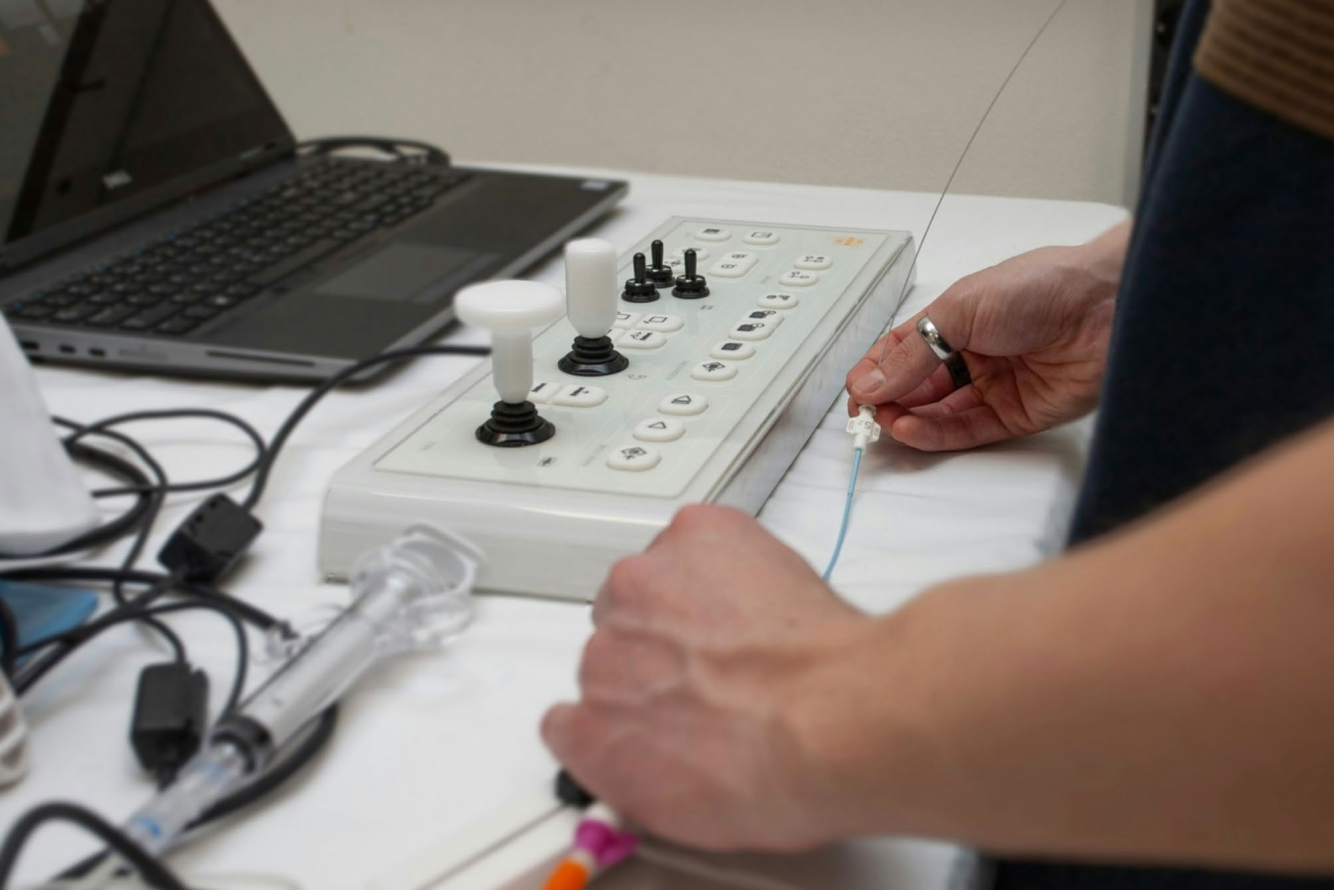
A study participant is engaged in a training session. The image shows the hands firmly gripping the controls of a simulator, adjusting the catheter and wire actively. The simulator with its buttons and joysticks and the simulated contrast injection is visible in the background.

Exercises were conducted on the same endovascular simulator (VIST G5 Mentice, Gothenburg, Sweden) by using Microsoft Windows (Version 10.0.19045) at the Endolab Benjamin Franklin Campus Charité. This advanced simulator, equipped with a 3D monitor, control joysticks, fluoroscopy and CINE pedals, and a device insertion model, emulates a catheter lab environment as shown in Figs. 2 and 3. Participants engaged in VIST Exercise ID 212 by Mentice (VIST Case file: gen_ii_a212_mainconfig.xml), a peripheral arterial occlusive disease scenario (in the Iliac and SFA Intervention 1.4.0 Module), involving the treatment of iliac and superficial femoral artery lesions. Preset instruments were.

- Crossover 8 Fr 90 cm Sheath.
- Crossover 4 Fr 90 cm selective catheter.
- 0.035-inch 300 cm J-Curve hydrophilic guidewire.
- Balloon dilation (iliac lesion): 40 mm length, 10 mm diameter ~ 8 atm pressure.
- Self-expanding stent (femoral artery): 120 mm length, 10 mm diameter.

**Fig. 4:**
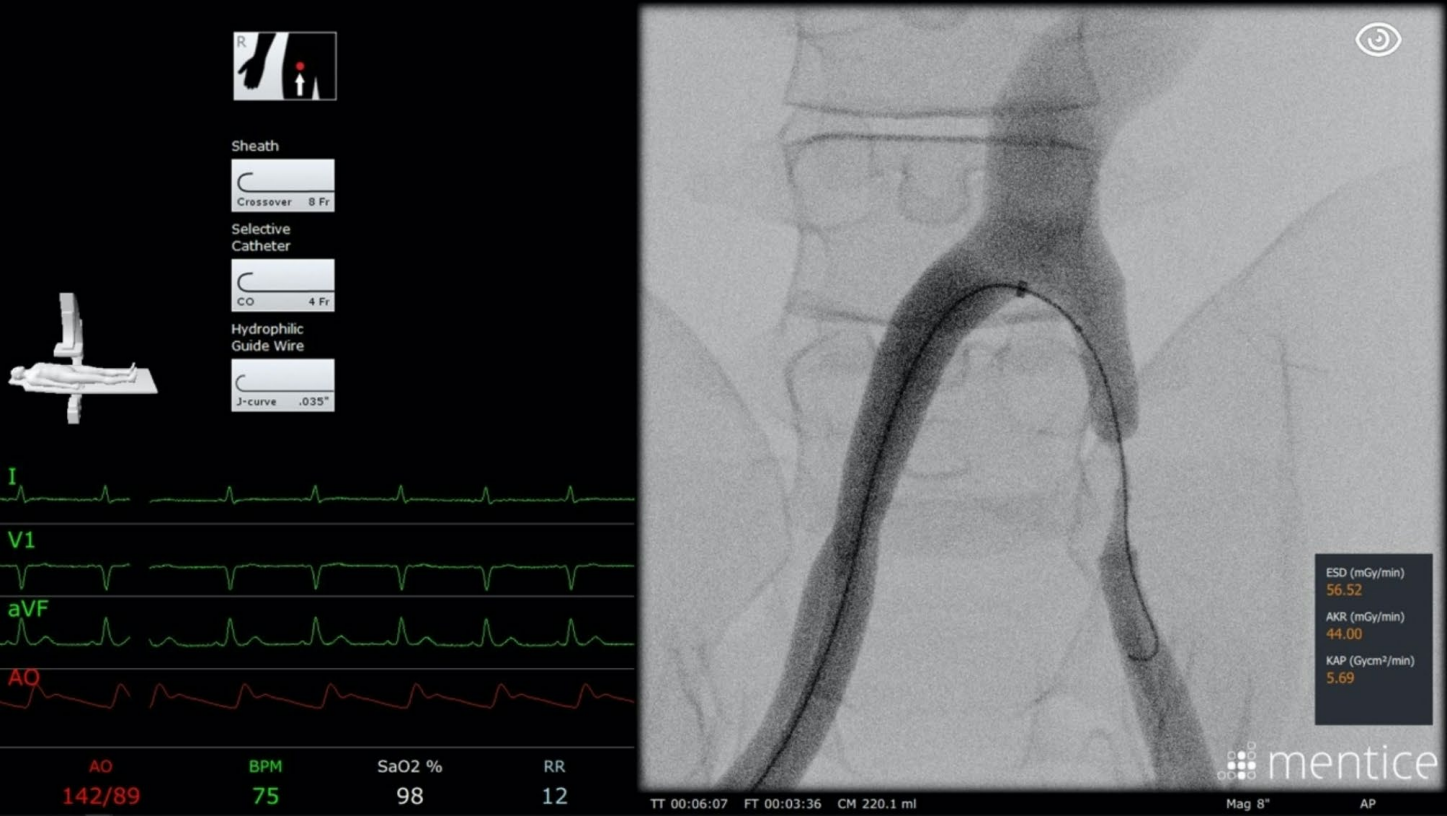
Screenshot of the simulator user interface during the treatment of a common iliac artery stenosis. The image shows the detailed interface with selected devices in the top left corner, highlighting the realistic and interactive elements of the simulation that aid in the training of angiographic procedures. The detailed and interactive interface helps participants familiarize themselves with actual procedural elements, leading to better preparation for real-world clinical scenarios.

The procedure started by introducing the sheath, selective catheter, and hydrophilic guidewire into the right femoral artery, through an existing opening in the simulator. The right femoral and iliac arteries, as well as the aortic bifurcation, were passed before reaching the first stenosis in the left iliac artery as shown in Fig. 4. The selective catheter was replaced by a balloon for a dilatation, and contrast medium was used to prove the dilatation. After exchanging back to the catheter, the left iliac common femoral and superficial femoral artery were explored to reach the second stenosis. This was treated by a stent substituted for the catheter as well. The intervention was finished by checking the success of the treatment with contrast medium in the superficial femoral artery and removing all instruments. The participants were allowed to use the amount of contrast medium and virtual fluoroscopy they needed to conclude the case successfully.

Training intervals varied (7–21 days) to discourage memorization by repetition (rote learning), thus fostering that recorded progress was indicative of practical skill enhancement. The training was repeated five times in total. Novices were encouraged to ask questions about the procedure and received guidance during each training session; however, there was no direct physical intervention by others. The session concluded upon the successful treatment of both lesions. Progress was assessed by recording virtual fluoroscopy time, which reflects proficiency and potential radiation exposure reduction. The study participants were unaware of which parameter served as a measure of improvement, allowing them to complete the exercises in an unbiased manner. The simulator software recorded pseudonymized data during exercises, including total time, fluoroscopy time, virtual radiation exposure, and further tool usage details.

Participants completed the questionnaire shown in Table 1 before and after completing the exercise to assess their experience and perception. The Likert scale (1-I do not agree at all to 5- I completely agree) gauged agreement with statements regarding skills, interest in interventional radiology, and simulator training impacts. Some questions were omitted from the analysis due to the pilot’s scope and sample size constraints.

**Table 1:** Pre- and post-training questionnaire.

Pre-exercise statements	
1	I have gained theoretical experience in the field of Interventional Radiology during my studies.
2	I assess my manual dexterity/finger skills as good.
3	I regularly play computer games.
4	Before starting my medical studies, I had completed training or studies in healthcare.
5	I regularly play a musical instrument.
6	I could imagine working professionally in interventional Radiology.
7	I am interested in the field of interventional Radiology.
8	I have already completed internships/clerkships in Radiology.
9	I consider my understanding of technical devices, such as computers, to be good.
Post-exercise statements	
1	Over the course of the exercises, my skills on the simulator have improved.
2	Through participating in the exercises, I have gained a better understanding of the field of interventional radiology.
3	The exercises on the simulator make me feel better prepared for the practical application of procedures on patients.
4	Training on the simulator has increased my interest in the field of interventional radiology.
5	Participating in the exercises helps me explain the field of interventional radiology to patients in an understandable way.
6	Simulator exercises enhance my understanding of Peripheral Arterial Disease.

### Statistical analysis

SPSS (29.0.1.1) software was utilized for data analysis. A Wilcoxon Signed Rank test for related samples assessed the primary hypothesis of reduced fluoroscopy time. Box-and-whisker plots depicted virtual fluoroscopy time data, highlighting distribution, central tendency, and variation. Therefore, average and median virtual fluoroscopy times were calculated individually for each exercise unit. The interquartile range (IQR), which is the range between the first and third quartiles, is representing the box as a measure of statistical dispersion. Maximum and minimum values are representing the whiskers of the plot. These measures provided insights into both central tendency and overall trends. The absolute virtual fluoroscopy times of the first and fifth units were compared to scale the magnitude of improvement. Additionally, relative comparisons within the participant group were made to identify consistent trends. The questionnaire’s categorical responses were analyzed using frequencies, percentages, and bar charts.

## Results

### Virtual fluoroscopy time reduction

**Fig. 5:**
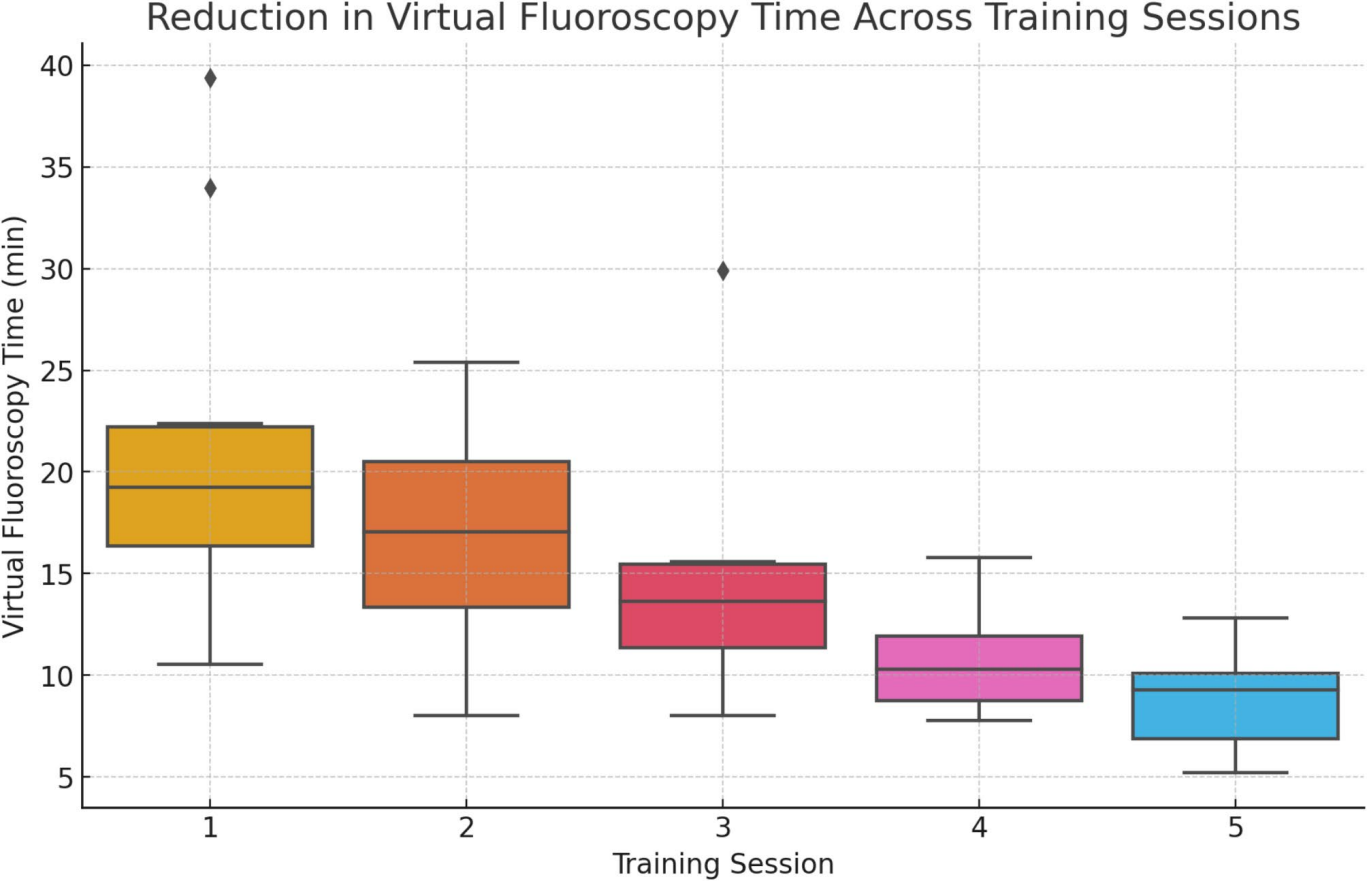
Box plot illustrating the reduction in virtual fluoroscopy time across the five training sessions. This figure demonstrates the median, interquartile range (IQR), and overall distribution of virtual fluoroscopy times, indicating a significant improvement in procedural proficiency among the participants. Key Finding: The consistent reduction in virtual fluoroscopy time, with a median decrease from 19.3 to 9.3 min, underscores the effectiveness of repetitive simulator training in enhancing procedural efficiency.

**Fig. 6:**
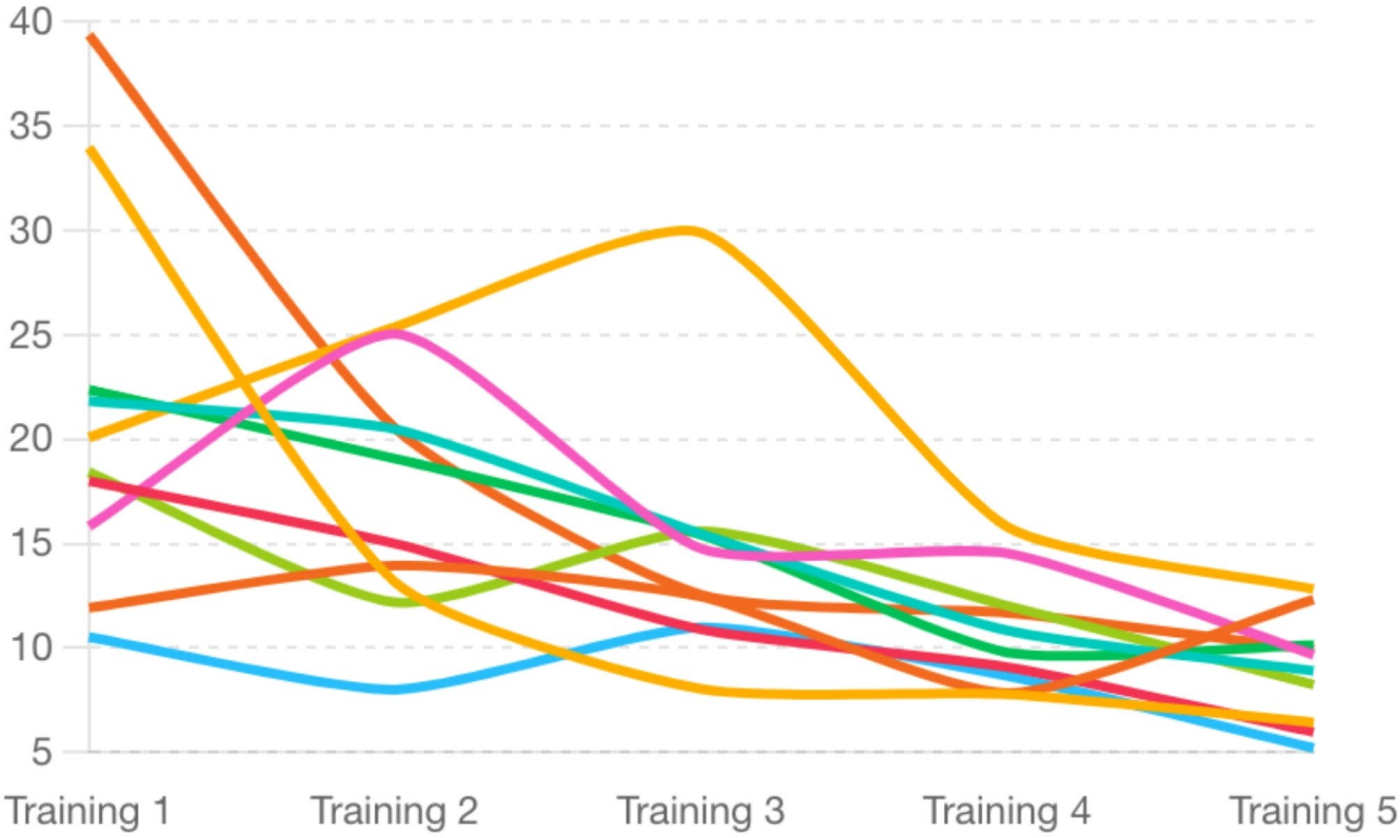
Line chart displaying the individual progress in virtual fluoroscopy time for each participant across five training sessions. The chart highlights the variability in learning curves and skill acquisition among participants.

Over the course of the five training sessions, the average virtual fluoroscopy time of the ten medical students constantly decreased (Table 2; Figs. 5 and 6). During the initial training session, the average (SD) virtual fluoroscopy time was 21.2 (9.0) minutes with a median (IQR) of 19.3 (16.4–22.2) minutes. Notably, the steepest mean reduction in virtual fluoroscopy time, 3.8 min, occurred between the third and fourth session. Between the other exercises, a consistent decrease in virtual fluoroscopy time, averaging 2.49 min per session, was recorded. Overall, there was an average reduction of 12.27 min (median of 11.11 min) in virtual fluoroscopy time between the first and the last training session. The resulting average final session time was 8.96 min with a standard deviation of 2.56 min. The time reduction from the first to the last training session is statistically significant (*p* = 0.007). The total time and radiation exposure were measured as well but correlate with the measured values for the virtual fluoroscopy time.

**Table 2:** Participant information and training results.

Participant	Training session 1 (min)	Training session 2 (min)	Training session 3 (min)	Training session 4 (min)	Training session 5 (min)	Percentual improvement (%)	Absolute improvement (min)
1	33.98	13.15	8.03	7.78	6.43	81.01	27.55
2	11.92	13.95	12.45	7.83	12.32	− 3.25	− 0.40
3	18.00	15.00	10.88	9.08	5.97	66.83	12.03
4	15.82	25.05	14.75	14.53	9.67	38.87	6.15
5	10.52	8.00	10.98	8.65	5.20	49.43	5.32
6	21.83	20.48	15.47	10.83	8.90	59.23	12.93
7	22.37	19.07	15.43	9.78	10.13	54.72	12.23
8	18.42	12.18	15.60	12.02	8.23	55.32	10.18
9	20.08	25.38	29.90	15.80	12.82	36.84	7.27
10	39.38	20.53	12.55	11.68	9.92	74.81	29.47

### Individual participant progress

The individual progress of participants differed within the participants’ group: notably, three participants exhibited an increase in fluoroscopy time during the second session compared to their initial session and again during the third session compared to the second. Two individuals showed an increase in time for the last session compared to the fourth. One participant’s time increased from 11.9 min in the first session to 12.3 min in the fifth. The highest percentual improvement was observed in a participant who decreased the virtual fluoroscopy time by 81.4% from 33.98 min in the first session to 6.43 min in the last.

### Questionnaires

Of the 10 participants, 4 were male and 6 were female. They were between 21 and 31 years old. Initial interest in Interventional Radiology was low, with only one participant considering it as a potential career path. Six were neutral, and three were disinterested. Despite this, 8 participants indicated a fundamental interest in IR, while two were neutral. However, by the end of the study, 80% of participants reported an increased interest after the simulator training, with 20% maintaining their initial neutral interest level. The practical skills and understanding of interventional radiology were perceived to have improved for all participants. Additionally, the majority felt better prepared for real-world interventions. Nine out of ten found the exercises beneficial in enhancing their ability to explain interventional radiology to patients and to understand Peripheral Artery Disease (PAD) more comprehensively.

### Correlation between questionnaire responses and training outcomes

Of all participants, only one had completed a radiology internship prior to the study and showed a 54.7% reduction in fluoroscopy time—just below the group average. Intriguingly, the participant with a regular video gaming habit achieved the most fluoroscopy time reduction at 81%, indicating a potential correlation between video gaming experience and proficiency in virtual simulations. However, the average reduction of virtual fluoroscopy time by non-video game players was only 48.09% lower compared to the participant engaging in video games.

## Discussion

This study investigated the efficacy of simulator-based training in enhancing the proficiency of medical students in interventional radiology endovascular procedures, particularly focusing on reducing virtual fluoroscopy time. Our findings suggest that simulator training can substantially decrease the time needed to perform simulated vascular interventions, indicative of improved procedural proficiency and potentially reduced radiation exposure in actual clinical practice.

The significant reduction in fluoroscopy time across training sessions underscores the value of repeated hands-on practice in developing technical skills. The steepest decline observed between the third and fourth session may suggest a learning curve where foundational skills and familiarity with the simulator interface become well-established, allowing for more efficient procedural execution. This shows that participants truly improved their skills and not only adapted quickly to the endovascular simulator itself.

Virtual fluoroscopy time was used as a parameter for recording and developing skills. This is reproducible, comparable, and a clear objective criterion. Furthermore, Jensen et al. showed that fluoroscopy time was the best parameter to evaluate the stage of achievement and is apparently well-suited for assessing performance^11^. Although the study involved cardiological interventions, it can be assumed that the result can be transferred to peripheral interventions.

As repeating the same cases shows an improvement in skills, this can potentially be transferred to the real clinical everyday life and could improve patient outcomes. Because of the improved skills, the time of the actual interventional radiology procedure and costs could be saved. The reduction in radiation time can be categorized as beneficial for both the staff and the patient^12^. Another advantage of the endovascular simulator is its ability to integrate real patient image data. This feature allows simulating and training for specific scenarios using actual patient data before performing the intervention in the real world and trying out new methods and instruments. Especially patients with particularly challenging anatomies and IR treatment procedures could benefit from this feature.

The variability in performance among participants highlights the personalized nature of learning processes. Some learners may experience initial challenges that temporarily impede performance improvements, as evidenced by the increased fluoroscopy times in the second and third sessions for some individuals. This underscores the need for tailored educational approaches that consider individual learning trajectories.

A notable shift in the participants’ interest in interventional radiology was observed post-training, with the majority expressing increased enthusiasm. This change could be attributed to the immersive experience provided by the simulator, offering students a glimpse into the specialty that they might not have had through traditional didactic education alone. Because of the forecasted personal shortage in IR, it is important to start arousing students’ interest and familiarizing them with IR early. Many students have an incomplete idea of what IR means and what an interventional radiologist does. For making IR more interesting, one could elevate the exposure of IR during medical education by implementing a rotation in IR or hands-on practice to improve understanding of the field for recruitment. These factors may correlate according to Nissim et al.^13^.

The correlation between participants’ backgrounds and their performance offers intriguing insights. For instance, the participant with prior radiology internship experience showed less improvement compared to the group average, which may indicate that some baseline familiarity with IR procedures could limit the perceived incremental benefit from simulator training. Conversely, the participant who frequently played video games exhibited the greatest improvement, suggesting that skills transferable from gaming such as hand-eye coordination, manual skills, and decision-making under pressure could enhance the learning curve in a simulated medical environment.

VR simulators offer the option to connect theoretical background with practical experience, by which understanding is consolidated. This would be one of the most effective ways to improve the intervenor’s skills and can be an option to enthuse students and young doctors for interventional radiology. Training with simulators boosts theoretical understanding and practical knowledge significantly more than traditional teaching without such technical equipment. According to educational psychologist Kolb, the fusion of situated learning, social learning, and practical-based learning is the most effective way of learning, which he describes as an experiential learning cycle^14^. Simulators can play a huge role in this cycle and improve students’ skills and, in conclusion, the quality of medical care.

Both skills—theoretical background knowledge and practical skills—are required to optimize patient care in everyday clinical practice. Better results can be achieved in the same amount of time. To truly improve that, it is necessary that simulator training results can also be transferred to real interventions on patients^15^. According to Nicholson et al., some simulators (like VIST by Mentice) can offer a comparable simulation of a real patient. Additionally, VR training has been proven to be a valid option for the implementation of teaching catheter interventions outside labs as a transfer of technology to clinical routines. Especially if used as an additional tool in clinical education but not as a substitution of traditional teaching in the education in interventional radiology^16–19^. Experienced interventionalists evaluate simulator training as a valid tool as well, even if they do not benefit significantly from simulator-supported teaching in direct comparison to beginners. Implementation of VR simulators seems most suitable for students and young professionals who focus on learning basic skills first. Simulators seem to be particularly suitable for enhancing this learning process^20–23^, but according to Cook et al. there is a structured curriculum needed to improve benefits^12^.

As learners engage in simulated medical scenarios, they transition from passive observation to active participation and eventually to interactive engagement with the simulation environment. This progression aligns with the “ICAP hypothesis” (Interactive, Constructive, Active, and Passive) proposed by Chi and Wylie (2014), which suggests that deeper levels of engagement lead to enhanced learning^24^.

### Limitations

This study has several limitations that should be considered when interpreting the findings. Firstly, the small sample size limits the generalizability of the results. Future studies with larger cohorts are needed to confirm these preliminary findings. Additionally, the study used virtual fluoroscopy time as the sole measure of proficiency, which does not account for other important factors such as accuracy, decision-making quality, or the ability to manage complications. Moreover, there was a selection bias, because participants might have had a basic interest or curiosity in IR, VR and simulators. This might influence the engagement and performance, although nine participants expressed their disinterest or neutral stance toward IR. Another limitation is the lack of investigation into the impact on theoretical knowledge and clinical decision-making, which could be addressed in future research. Finally, the study did not account for the potential influence of prior experiences, such as regular video gaming, on the learning outcomes, although this aspect showed interesting trends that warrant further exploration in larger studies.

## Conclusion

Simulator training emerges as a promising tool in medical education, offering a less risky platform for students and medical professionals to refine their skills and awaken interest in specialties like interventional radiology. It also hints at the potential for certain extracurricular activities, to contribute beneficially to the acquisition of medical procedural skills. The significant reduction in virtual fluoroscopy time observed across training sessions underscores the effectiveness of repeated practice in skill development. This improvement, coupled with the participants’ increased interest in interventional radiology post-training, suggests that simulator-based education can have a positive impact on both skill acquisition and career aspirations.

As technology continues to evolve, the role of simulation in medical training is expected to become more pronounced, warranting continued investigation into its most effective implementations. By identifying trends and correlations in learner performance, educators can tailor training programs to meet the diverse needs of medical students, possibly improving patient care outcomes in interventional radiology and beyond.

## Data Availability

The datasets used and/or analyzed during the current study are available from the corresponding author on reasonable request.
